# “I Want, Therefore I Am” – Anticipated Upward Mobility Reduces Ingroup Concern

**DOI:** 10.3389/fpsyg.2017.01451

**Published:** 2017-08-28

**Authors:** Marion Chipeaux, Clara Kulich, Vincenzo Iacoviello, Fabio Lorenzi-Cioldi

**Affiliations:** ^1^Social Psychology, Section of Psychology, Faculty of Psychology and Educational Sciences, University of Geneva Geneva, Switzerland; ^2^Social Psychology, Psychology, Faculty of Behavioural and Social Sciences, University of Groningen Groningen, Netherlands

**Keywords:** multiple social identities, social mobility, status inconsistency, ingroup concern, identification

## Abstract

Empirical findings suggest that members of socially disadvantaged groups who join a better-valued group through individual achievement tend to express low concern for their disadvantaged ingroup (e.g., denial of collective discrimination, low intent to initiate collective action). In the present research, we investigated whether this tendency occurs solely for individuals who have already engaged in social mobility, or also for individuals who psychologically prepare themselves, that is ‘anticipate’, social mobility. Moreover, we examined the role of group identification in this process. In two studies, we looked at the case of ‘frontier workers’, that is people who cross a national border every day to work in another country where the salaries are higher thereby achieving a better socio-economic status than in their home-country. Study 1 (*N* = 176) examined attitudes of French nationals (both the socially mobile and the non-mobile) and of Swiss nationals toward the non-mobile group. As expected, results showed that the mobile French had more negative attitudes than their non-mobile counterparts, but less negative attitudes than the Swiss. In Study 2 (*N* = 216), we examined ingroup concern at different stages of the social mobility process by comparing the attitudes of French people who worked in Switzerland (mobile individuals), with those who envisioned (anticipators), or not (non-anticipators), to work in Switzerland. The findings revealed that anticipators’ motivation to get personally involved in collective action for their French ingroup was lower than the non-anticipators’, but higher than the mobile individuals’. Moreover, we found that the decrease in ingroup concern across the different stages of social mobility was accounted for by a lower identification with the inherited ingroup. These findings corroborate the deleterious impact of social mobility on attitudes toward a low-status ingroup, and show that the decrease in ingroup concern already occurs among individuals who anticipate moving up the hierarchy. The discussion focuses on the role of the discounting of inherited identities in both the anticipation and the achievement of a higher-status identity.

## Introduction

Individuals are members of inherited social groups defined, for instance, by their gender, ethnicity, or age. They belong simultaneously to more malleable categories qualified by their educational and professional achievements. The present work is interested in how individuals cope with such multiple group memberships, in particular when these memberships are associated with different value and prestige (i.e., social status). Nowadays, most societies are still organized around a hierarchical principle of distribution of resources and power, creating and reinforcing economic, cultural, and political inequalities. Some groups are associated with a high social status whereas others with a low one (Tajfel and Turner, 1986; Sidanius and Pratto, 1999). Nevertheless, while in traditional societies the various group memberships tended to be aligned in status (Lenski, 1954), the stronger social fluidity of contemporary societies leads individuals to belong to multiple groups of conflicting status. For example, individuals from disadvantaged inherited backgrounds (e.g., women, ethnic minorities) may achieve higher status through professional attainments. The present research seeks to better understand the socio-psychological processes at play when individuals are confronted with such status inconsistency due to upward mobility. We first consider how they cope with the contradicting demands arising from such multiple group memberships, by investigating their concern for the inherited low-status group members who did not achieve social mobility (Study 1) and more generally toward the inherited low-status ingroup (Study 2). We then investigate whether the anticipation of social mobility already leads to a decreased ingroup concern.

Although being hierarchically organized, modern societies are characterized by an ideal of meritocracy that leads people to believe that personal investment and efforts are main causes of success (McCoy and Major, 2007). People are encouraged to focus on their personal trajectory and to engage in individual strategies in order to improve their social standing and to achieve self-worth (Tajfel and Turner, 1986; Wright, 2001). The social mobility strategy, as defined by social identity theory (SIT: Tajfel and Turner, 1986), describes individuals who suffer from the low status associated with their group membership and decide to quit their group to join a better valued one. However, although clear-cut scenarios can be designed in the laboratory in order to make salient one specific membership, the study of identities in real life is more complex. Most often, individuals are confronted with contexts in which several of their group memberships are salient. Moreover, we argue that the possibility to leave a social group for another drastically varies depending on the nature of the group memberships. Whilst some group memberships are achieved by individuals throughout their lives (e.g., professional occupation, political affiliation), other groups are imposed from birth and are thus inherited in quality (e.g., gender, ethnicity). For individuals who are members of low-status achieved groups, the social mobility process can effectively occur as they move from one group to another. An illustration is an individual’s attempt to quit their employee status by moving up the social ladder and becoming a manager. However, when the low-status membership is inherited, this status is quite impermeable, meaning that the individual has little power to modify it. For instance, a woman cannot easily change her sex, but she can focus on her professional standing and become a manager (Ellemers, 2001; Derks et al., 2016, for a review).

Past research has shown that there is a tendency toward status crystallization, meaning that the probability to achieve a high-status membership is greater for members of high-status inherited groups than for members of low-status inherited groups (Lenski, 1954; Bourdieu, 1984). Nonetheless, societies have become increasingly fluid over the past decades, notably because of social and political movements (e.g., feminism, human rights movements) which have contributed to break societal barriers. A product of these more fluid societies is the increasing number of individuals experiencing status-inconsistent identity configurations. According to Lenski (1966), individuals who are simultaneously members of low- and high-status groups experience a psychological tension derived from their motivation to improve their social identity while still belonging to a low-status group. In line with this idea, Wright and Taylor (1999) showed that low-status group members who succeeded as a token felt more negative emotions than individuals who succeeded in a non-discriminatory context. This means that, when being simultaneously members of low- and high-status groups, individuals face contradicting social expectations. Indeed, such expectations (e.g., stereotypes) differ to a great extent according to group status (Fiske et al., 2002; Kervyn et al., 2009). Moreover, while high-status groups promote norms and values related to independence, individualism, and self-fulfillment, low-status groups convey norms and values that promote interdependence and solidarity among its members (Lorenzi-Cioldi, 1988, 2009). Thus, by conforming to the norms of one of their memberships, individuals in status-inconsistent identity configurations deviate from the norms of their other membership, and expose themselves to various forms of social punishments. As an illustration, female managers may be punished for enacting agentic behaviors, because these behaviors contradict the female stereotype despite the fact that agency is expected for the professional role (Rudman and Glick, 2001).

Consistent with the motive to achieve self-worth as posited in SIT, individuals who possess multiple social identities have “a natural tendency to think of themselves in terms of that status or rank which is highest, and to expect others to do the same” (Lenski, 1966, 87). Providing evidence of Lenski’s reasoning, a series of studies conducted by Derks and colleagues showed that, among women who achieved a high-responsibility professional role, those who reported low levels of gender identification and who reported having experienced gender discrimination tended to describe themselves as more similar to the high-status group, compared to women high in gender identification and/or women having experienced low gender discrimination. In this way, they portrayed themselves using more masculine traits (the characteristics of the high-status group), while using the same amount of feminine traits as other women (Derks et al., 2011a,b). In addition, research showed that female faculty rated their male Ph.D. students more favorably than their female Ph.D. students, whilst no difference was observed among the male faculties’ evaluations (Ellemers et al., 2004). Of interest, the same pattern of results was observed among Hindustanis in the Netherlands who self-described as more Dutch when lowly identified with their ingroup and when having experienced discrimination (Derks et al., 2015). Lack of ingroup support has also been reported from the perspective of achieved low-status individuals. Research showed that female employees and Non-White employees felt less support from ingroup supervisors (i.e., female and Non-White supervisors) than from outgroup supervisors (i.e., male or White supervisors) in organizations with an adverse diversity climate (Paustian-Underdahl et al., 2017). Such parallel findings across social categories suggest that low ingroup concern among socially mobile women is not specific to gender, and that it can be broadly attributed to the status dynamics between different group memberships. Providing evidence to this reasoning, Kulich et al. (2015) compared the ingroup concern of low-status inherited group members who had successfully engaged in social mobility to their congeners who had not. In their research, the authors observed that mobile members of different social categories of inherited low-status groups (e.g., Afro-Americans, immigrants, and women) expressed greater hostility and lesser support for the inherited low-status group compared to non-mobile members. Taken together, these findings suggest that individuals who experience a status-inconsistent identity configuration describe themselves as more similar to the achieved high-status group and are less supportive of the low-status inherited group.

An important issue is the kind of motivation that leads to such ingroup unsympathetic attitudes. Predictions derived from SIT (see Ellemers, 2001) would explain the lack of ingroup concern by a decrease in ingroup identification. However, Kulich et al.’s (2015) findings revealed that identification with the inherited group did not play a role, as mobile and non-mobile participants were similarly identified with their inherited group. This suggests that, despite their self-distancing from the inherited group on the attitudinal dimension and their stronger counter-stereotypical self-descriptions, the mobile remained identified with this group, and also similarly self-described on the dimension that is stereotypical of it. In addition, the authors found that the lack of support for the inherited ingroup was accounted for by an increased identification with the achieved high-status group. Mobile individuals identified more strongly with their achieved group than the non-mobile. Such pattern suggests that individuals with multiple identities do not necessarily disengage from their low-status group. They may have to cope with the simultaneous presence of several identities and thus the coping strategies on the attitudinal, self-evaluation, and self-categorization levels are not aligned. For example, the disparagement of the inherited low-status ingroup may be motivated by an effort to become accepted in the new high-status group (Wright and Taylor, 1999), while keeping the ties with the low-status inherited group. Indeed, the conflicting nature of their identity configuration becomes evident in the contrasting phenomena of identification and simultaneous negative attitudes toward the low-status ingroup. Research is thus needed to identify the specifics of this assimilation process which does not appear to influence to the same extent the different dimensions of analysis (e.g., attitudes, identification).

One way to look at this assimilation process is to compare the attitudes of mobile individuals to the attitudes of the high-status group members. Indeed, considering the motivation of low-status groups’ members to enhance the positivity of their social identity, one may expect that mobile individuals adopt similar attitudes as those of high-status groups’ members, that is, members of groups that mobile individuals joined through their social mobility. This could be considered as a strategy to increase their chances to be accepted in the new group by showing to its members that they do not consider themselves as members of the low-status (out)group anymore (Merton, 1968). Indeed, Van Laar et al. (2014) showed that members of the high-status group offered support to the mobile only if they perceived that the latter were not behaving in a manner that was prototypical of their low-status group. Nevertheless, we also know from the social identity perspective that social groups’ members need to feel their membership not only as positive, but also as distinct (Tajfel and Turner, 1986). Thus, to achieve this feeling of distinctiveness, individuals tend to express ingroup bias. This bias describes the tendency of individuals to show more negative attitudes toward outgroup members, compared to ingroup members, especially when social categorization is salient (Mullen et al., 1992). This may help to ensure the distinctiveness of their group membership, but also indirectly to increase their self-esteem (see for example, Brown, 2000). In sum, it appears more reasonable to think that in order to maintain the distinctiveness of their membership, members of the high-status group will still express more negative attitudes toward the low-status group members, than the mobile individuals, thus preventing a complete assimilation of mobile individuals which would threaten their distinctiveness.

A second way of looking at this assimilation process is to wonder whether social mobility in itself is related to a lack of ingroup concern, or whether this relationship is already present among individuals who merely aspire to undertake social mobility. Indeed, Merton (1968) suggests that social mobility is often preceded, or facilitated, by the expression of positive attitudes toward the group to which the individual seeks to belong to. He theorized this process as an *anticipatory socialization* which contributes to increase the probability of successful individual mobility, as well as the integration in the new group once joined. In line with this idea, research by Ellemers et al. (1993) showed that, when people find themselves in a permeable intergroup context, they tend to act in order to defend their individual interests rather than the interests of their group (see also Wright et al., 1990). Ellemers et al. (1990) further showed that, in a permeable context where individual mobility is facilitated, competent individuals strongly identify with the high-status group. Thus, on the basis of this literature, we expect different levels of ingroup concern between the non-mobile who do not strive for social mobility and the non-mobile who do. In conformity with Merton’s (1968) suggestion, anticipators of social mobility should reduce their ingroup concern in order to enhance their chances to achieve the mobility. Finally, even if anticipators should reveal a lower concern than the non-anticipators, we expected them to still be more concerned than mobile individuals. The latter, who successfully achieved social mobility, should be motivated to maintain their distinctiveness by distancing even more strongly from their low-status inherited ingroup.

### The Present Research

In order to examine the identity management strategies in dual identity configurations, we conducted two correlational studies. Our target group consisted of French nationals living in areas around the Swiss border. As the costs of living are higher in this region than the average costs in France, many French from this region attempt to join the Swiss work-force which grants a number of financial and symbolic advantages: The unemployment rate in Switzerland is almost half (5.1%, FSO, 2014) of the rate in France (9.9%, INSEE, 2014), and the median salary in Switzerland (5,560 euros) is three times higher than the median salary in France (1,712 euros). We took advantage of this natural setting and compared these socially mobile French ‘frontier workers’, who achieved a considerably higher socio-economic standing, with the non-mobile French who worked in France.

Our first aim was to test whether mobile individuals (i.e., French frontier workers) are less concerned with the achieved low-status group that they have left (i.e., French workers in France; see Study 1) and with their inherited ingroup as a whole (i.e., French people who live in border regions of Switzerland; see Study 2), compared to non-mobile individuals (i.e., French workers in France). Moreover, we looked at the relevant inherited high-status outgroup (i.e., Swiss workers in Switzerland), who are granted consistency between their inherited and achieved memberships (see Study 1). This design bore the opportunity to assess the extent to which mobile individuals assimilate to the high-status group. Finally, we examined whether the actual achievement of social mobility is a necessary condition to undermine ingroup concern, or whether the mere prospect of undertaking social mobility is sufficient to do so. This was done by measuring non-mobile participants’ willingness to engage in social mobility (Study 2). From these general goals, we derived the following two hypotheses:

Hypothesis 1 (tested in Study 1) predicts a linear effect of social mobility on the concern for the low-status achieved group. More specifically, we expect non-mobile individuals to express more concern than the mobile individuals, who in turn should express more concern than the high-status inherited group members. We thus aimed to demonstrate that mobile individuals, who achieved a high-status position through individual mobility, express a lower concern for the fate of their inherited group members who did not succeed individually, and that they have the willingness to assimilate with the high-status group. Moreover, we sought to highlight that the high-status group’s members can feel threatened by mobile individuals and should therefore express an even lower concern. This would safeguard their ingroup distinctiveness.
Hypothesis 2 (tested in Study 2) predicts a linear effect of the stages of social mobility (non-anticipators, anticipators, mobile individuals) on the concern for the inherited low-status group. More specifically, we expect the concern for the low-status inherited ingroup to be highest among non-mobile individuals who do not wish to undertake mobility (i.e., non-anticipators), moderate among non-mobile individuals who strive for mobility (i.e., anticipators), and lowest among individuals who have succeeded in their mobility (i.e., mobile) – the latter being motivated to claim their distinctiveness in the face of the anticipators. Indeed, even if anticipators have a strong desire to improve their social status, they are still part of the non-mobile group (i.e., by being simultaneously members of low-status achieved and inherited groups), and for this reason, they should still express a higher concern for their inherited membership compared to mobile individuals who can focus on their high-status achieved membership in order to reduce the identity threat associated with their low-status inherited membership.

In addition, we aimed to investigate the mechanisms underlying these attitudinal differences. Although the social identity perspective leads to the expectation that self-ingroup distancing derives from a lower identification with the inherited group, this has not been found in past research (Kulich et al., 2015). We believe that different levels of inherited ingroup identification could have been concealed in previous studies because such studies only looked at non-mobile individuals without making the distinction between anticipators and non-anticipators of social mobility. To move a step forward, our research considered social identification with the inherited group among these two non-mobile subgroups. From this, follows our next hypothesis:

Consistent with assumptions derived from SIT, Hypothesis 3 (tested in Study 2) predicts that the lower ingroup concern among anticipators of social mobility (as compared to non-anticipators) and even lower concern among mobile individuals should be explained by a lower identification with the low-status inherited group.

### Study 1

Study 1 tested H1, which predicts a linear effect of social mobility on concern for the low-status achieved group. More specifically, we examined this concern among French workers in France (i.e., the non-mobile individuals), French workers in Switzerland (i.e., the mobile individuals), and Swiss workers in Switzerland (i.e., the members of the high-status group).

In addition, we also explored the identification patterns associated with these different categories.

#### Method

##### Participants

A total of 176 participants (122 women and 54 men, *M*_age_ = 34.53, *SD*_age_ = 9.23, ranging from 19 to 62 years old) were recruited through social networks and were asked to complete an online questionnaire. One-hundred and fifteen participants were French and 61 were Swiss.

##### Materials and measures

Participants indicated their citizenship and were then presented with a short excerpt in which France and Switzerland were compared on several domains, such as employment rates and average wages. The aim of this introductive part was to emphasize the current socio-economic status gap of the two national groups. Participants were then asked to answer several items, which are listed below in the chronology of their occurrence.

###### Social mobility

The social mobility variable distinguished between three groups of participants. French working in France were the *non-mobile* group (*n* = 43). These participants are characterized by their relative low inherited status (i.e., French as compared to Swiss nationals) along with a low achieved status (i.e., they work in France). French nationals working in Switzerland were the *mobile* group (*n* = 72). These participants are characterized by a low inherited status combined with a high achieved status (i.e., French working in Switzerland). Finally, Swiss nationals working in Switzerland (*n* = 61) were the high-status group, characterized by high inherited and achieved statuses.

###### Identification with the inherited and the achieved groups

Identification, with the inherited and the achieved groups, was assessed with the 10 items of the self-investment dimension of the hierarchical model of ingroup identification (Leach et al., 2008). Sample items are “I feel a bond with [Ingroup]”, “I’m glad to be [Ingroup]”, or “I often think about the fact that I am [Ingroup]” (1 *fully disagree* to 7 *fully agree*). First, participants were asked to answer these items for their inherited group (i.e., “French people in general” or “Swiss people in general”). The reliability of this scale was satisfactory for both targets (respectively, α = 0.90; *M* = 4.77, *SD* = 1.36 for the French, *n* = 115, and α = 0.89; *M* = 5.42, *SD* = 1.03 for the Swiss, *n* = 61). Second, they were asked to answer these items for their achieved group (i.e., “workers in France” or “workers in Switzerland”). The reliability of this scale was also satisfactory for both targets (respectively, α = 0.84; *M* = 4.43, *SD* = 1.16 for the workers in France, *n* = 43, and α = 0.84; *M* = 5.29, *SD* = 0.96 for the workers in Switzerland, *n* = 133).

###### Concern for the low-status achieved group

We measured participants’ motivation to engage in social action aimed at improving the situation of French nationals who lived in border regions of Switzerland and worked in France. We measured support for both personal involvement and group involvement in social action because this allowed to capture potential psychological distancing from the group as a result of a simultaneous expression of high support for group involvement and low motivation to get personally engaged. These measures were taken on a 7-point scale, from 1 *not at all* to 7 *totally*.

Support for *group involvement* was assessed with two items (e.g., “French people who work in France and live in border areas should fight collectively for financial compensation for the difference they face between the cost of living and the level of their wages”, *r* = 0.64, *p <* 0.001; *M* = 4.67, *SD* = 1.79). *Personal involvement* was assessed with two items measuring participants’ motivation to get personally involved in social action (e.g., “I would be willing to sign a petition to call for more economic support for French people who work in France and live in border regions”, *r* = 0.55, *p <* 0.001; *M* = 3.78, *SD* = 1.87).

We also introduced a direct measure of *concern for the low-status achieved group* with the single item: “I feel concerned by the fate of French people living in border areas of Switzerland and working in France” (1 *not at all* to 7 *totally*; *M* = 4.19, *SD* = 1.95).

###### Socio-demographic information

Finally, participants indicated their gender, their professional status (with in general 67.6% employees, 8.5% entry-level managers, 9.1% middle managers, 8% senior managers and 6.9% missing data), and their age (*M* = 34.53, *SD* = 9.23; ranging from 19 to 62 years old). We also measured the subjective social status of their occupation with two items (i.e., “To what extent do you think that your professional occupation is valued – and – prestigious in society?”) (1 *not at all* to 7 *totally*, *r* = 0.46, *p <* 0.001, *M* = 3.98, *SD* = 1.34). Moreover, we measured the perceived status of working in Switzerland and France with two items (“To what extent do you think that working in France/Switzerland is valorizing?”, 7 point-scale from 1 *not at all* to 7 *totally*, with, respectively, *M* = 3.34, *SD* = 1.62 for the French, and *M* = 5.29, *SD* = 1.22 for the Swiss).

#### Results

##### Preliminary analyses

We performed several analyses in order to detect potential differences on relevant socio-demographic indicators between the three groups of participants. First, we looked at gender, as the occupational gender divide may lead to men and women occupying professions that differ in type and status (e.g., Charles and Grusky, 2004). Chi-square analysis showed that men and women were similarly distributed across the three mobility groups, χ^2^(2, *N* = 176) = 3.34, *p* = 0.18. Moreover, the three groups did not differ in professional status, χ^2^(6, *N* = 164) = 8.80, *p* = 0.18. ANOVA of the continuous variable measuring the subjective social status of the occupation, with social mobility as a between-participants factor, revealed no effect, *F*(2,172) = 0.69, *p* = 0.50, $\eta_{p}^{2}$ = 0.16. We also tested if the status that the three investigated groups attributed to working in France and in Switzerland differed. In a repeated-measures ANOVA with the two status items and the three participant groups as a between-participants factor, we observed that all groups of participants rated working in Switzerland as more valued than working in France, *F*(1,171) = 144.49, *p <* 0.001, $\eta_{p}^{2}$ = 0.46. Furthermore, this effect was qualified by a significant interaction effect between the two factors, *F*(2,171) = 4.11, *p* = 0.018, $\eta_{p}^{2}$ = 0.05. Although the French workers also believed that working in Switzerland was more valued than working in France (*p <* 0.001), pairwise comparisons revealed that the French working in France attributed a higher value to working in France than the other two groups (*p*s < 0.04). Finally, we tested for age differences in the three mobility groups and observed a marginal effect, *F*(2,173) = 2.53, *p* = 0.08, $\eta_{p}^{2}$ = 0.03. Pairwise comparisons showed that French workers in France (*M* = 31.81, *SD* = 6.94) were younger than French (*M* = 35.28, *SD* = 9.04, *p* = 0.051) and Swiss (*M* = 35.57, *SD* = 10.54, *p* = 0.04) workers in Switzerland. No difference was observed between the two groups working in Switzerland (*p* > 0.85). In light of this unexpected finding, participant age was entered as a covariate in all of the following analyses^1^.

##### Hypotheses testing

###### Concern for the low-status achieved group

In order to test H1 which predicts a linear effect of social mobility on concern for the low-status group, we computed two orthogonal contrasts with the social mobility variable. The first contrast (C1) opposed the French workers in France (i.e., non-mobile), coded -1, to the Swiss (i.e., high-status group members), coded 1, with the French workers in Switzerland (i.e., mobile) coded 0, lying in between these groups. The residual contrast (C2) opposed the French workers in Switzerland, coded -2, to the two others groups, the French workers in France and the Swiss, both coded 1. H1 predicted a significant effect of C1 but not C2, thus highlighting a linear effect of the social mobility variable (Judd et al., 2011).

We performed a repeated-measures ANCOVA with personal and group involvement measures of social action as a within-participant factor, the two orthogonal contrasts as between-participants factors, and age as a covariate. The findings showed a significant main effect of involvement in social action, *F*(1,172) = 59.57, *p <* 0.001, $\eta_{p}^{2}$ = 0.26, such that personal involvement (*M* = 3.78, *SD* = 1.87) was lower than group involvement (*M* = 4.67, *SD* = 1.79). The analysis further produced an interaction between involvement in social action and C1, *F*(1,172) = 15.02, *p* > 0.001, $\eta_{p}^{2}$ = 0.08, showing that C1 had a significant effect on the motivation to get personally involved in social action, *t* = -3.61, *p <* 0.001, but did not impact significantly the group dimension, *t* = -0.67, *p* = 0.50. As the interaction between involvement in social action and C2 was not significant, *F*(1,172) = 0.34, *p* = 0.56, $\eta_{p}^{2}$ = 0.002, the C1 effect can be interpreted as linear effect. As predicted in H1, Swiss participants (*M* = 3.20, *SD* = 1.81) were less motivated to get personally involved in social action for the French working in France compared to the French workers in France themselves (*M* = 4.53, *SD* = 1.83, *t* = -3.61, *p <* 0.001) with the French workers in Switzerland (*M* = 3.82, *SD* = 1.81) situated between these two groups. The interaction between involvement measures and age was not significant, *F*(1,172) = 0.89, *p* > 0.34, $\eta_{p}^{2}$ < 0.01.

We then conducted an ANOVA on the single item of concern for the low-status achieved group, with the two orthogonal contrasts as between-participants factors and age as a covariate. The pattern of results was quite similar to the one observed for personal involvement in social action. The analysis showed a significant effect of C1, *F*(1,172) = 37.75, *p <* 0.001, $\eta_{p}^{2}$ = 0.18, and a marginal effect of C2, *F*(1,172) = 3.14, *p* = 0.078, $\eta_{p}^{2}$ = 0.018. Thus, as predicted in H1, we observed a linear tendency showing that Swiss participants (*M* = 3.03, *SD* = 1.95) expressed a lower concern for the low-status achieved group compared to the non-mobile French (*M* = 5.16, *SD* = 1.97). The French workers in Switzerland (*M* = 4.60, *SD* = 1.81) were situated between these two groups, and were closer to the French workers in France than to the Swiss.

###### Identification with the inherited and the achieved groups

**FIGURE 1:**
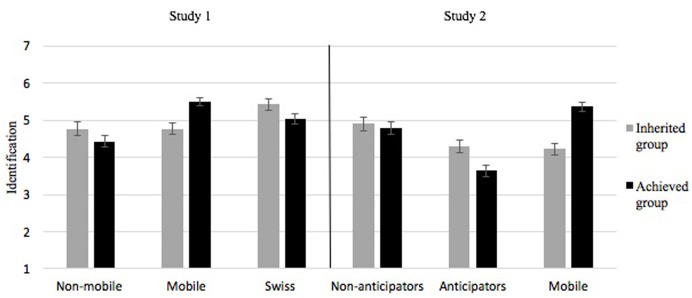
Means of inherited and achieved identification (Study I left, Study 2 right). Error bars indicate standard errors.

In order to investigate the identity patterns of the non-mobile, the mobile and the high-status group members, we performed a repeated-measures ANCOVA with inherited versus achieved identification as a within-participant factor, the three participant groups as between-participants factor (i.e., corresponding to the social mobility variable), and age as a covariate. Results revealed an interaction between group and identification, *F*(1,172) = 18.61, *p <* 0.001, $\eta_{p}^{2}$ = 0.18 (means are presented in **Figure 1**, left panel). Pairwise comparisons first indicated that mobile participants identified more strongly with their achieved group than the Swiss (*p* = 0.01) and the non-mobile French (*p <* 0.001), while the Swiss identified more strongly with their achieved group than the non-mobile participants (*p* = 0.002), *F*(2,172) = 15.47, *p <* 0.001, $\eta_{p}^{2}$ = 0.15. Second, we also observed that the Swiss identified more strongly with their inherited group than non-mobile (*p* = 0.009), and mobile French (*p* = 0.003), while no differences were observed between these two last groups (*p* = 0.99), *F*(2,172) = 5.60, *p <* 0.01, $\eta_{p}^{2}$ = 0.06. We then investigated this interaction by focusing on the difference between identification with the inherited group and identification with the achieved group. Findings revealed that mobile participants identified more strongly with the achieved than the inherited group, *F*(1,172) = 27.68, *p <* 0.001, $\eta_{p}^{2}$ = 0.14, while the Swiss and the non-mobile French showed a reversed pattern and identified more strongly with the inherited than the achieved group, with respectively, *F*(1,172) = 6.60, *p* = 0.01, $\eta_{p}^{2}$ = 0.04 (for the Swiss), and *F*(1,172) = 3.69, *p* = 0.056, $\eta_{p}^{2}$ = 0.02 (for the non-mobile French).

#### Discussion

Consistent with Hypothesis 1, Swiss participants reported lower concern and lower motivation to get personally involved in social action for the French working in border regions of Switzerland, compared to the French working in these regions themselves. Moreover, the attitudes of the frontier workers (i.e., mobile individuals) were situated in between these two groups’ attitudes. Thus, we observed that even if the mobile French appeared to take distance from the low-status achieved group, they did not fully assimilate to the Swiss high-status group members. Indeed, they still expressed a higher concern and a higher motivation to get involved in social action compared to what reported the Swiss participants. These discrepancy between French mobile and Swiss participants can be interpreted through the lens of SIT (Tajfel and Turner, 1986). More specifically, by considering that the Swiss constituted an outgroup on both identity dimensions (i.e., inherited and achieved), it is expected that they show less support for the French, as compared to the French (either mobile or non-mobile). From the perspective of mobile individuals, French workers in France also belong to an outgroup, but on the achieved dimension exclusively. Consistent with previous research (e.g., Kulich et al., 2015), these findings highlight the negative impact of social mobility on the attitudes toward the ingroup. They also provide evidence that the assimilative dynamic toward the high-status group can be a consequence of the social mobility process. Indeed, we observed that even if French mobile participants effectively expressed less concern than their non-mobile counterparts, they still appeared more concerned than the high-status group members (i.e., the Swiss). As discussed in the Introduction, we argue that this attitudinal difference between French mobile and Swiss participants may also have been due to a motivation of Swiss participants to maintain their distinctiveness, potentially threatened by the arrival of mobile individuals.

Of interest, we observed different degrees of involvement in social action for the low-status achieved group on the individual and the group level. Although we found the predicted negative impact of social mobility on the motivation to get personally involved in social action, we did not observe any differences on the motivation for group involvement. These findings suggest that individuals, regardless of their status, acknowledged the disadvantaged conditions endured by the French working in border regions of Switzerland, and that they were favorable toward group involvement in social action. If we consider the normative pro-egalitarian context of contemporary societies, we can apprehend such a support for group involvement as a socially valued opinion resulting in a shared conformism to social norms.

Concerning the identification dimension, we observed a significantly higher identification with the achieved group among mobile French and Swiss workers compared to non-mobile French workers. Consistent with Kulich et al. (2015), this result indicates that mobile participants clearly focus on their achieved higher-valued identity, and distance themselves from their low-status group, as it was also observed on the attitudinal dimension. It is also consistent with the literature showing that individuals identify more strongly with ingroups that are more socially valued (e.g., Ellemers et al., 1990; Roccas, 2003). Moreover, still in line with the findings of Kulich et al. (2015), we did not observe any difference in the identification with the inherited group between mobile and non-mobile participants. This suggests that the mobile keep their ties with their inherited group and manage their social mobility through an increase in the identification with the new high-status achieved group. Nevertheless, as we claimed in the Introduction, we believe that the absence of an effect of the identification with the inherited group may be due to the fact that the non-mobile group is quite heterogeneous in the group members’ desire to engage in a mobility in the future. Study 2 will address this issue. Finally, comparison of the inherited identity patterns of the Swiss nationals and the mobile individuals revealed a higher identification with the inherited identity by the Swiss compared to the two French groups. This is not surprising as the Swiss have a more positive inherited identity in this intergroup context. Moreover, we observed that the Swiss revealed a preference for their inherited identity as compared to their achieved identity, which was the exact opposite of the pattern observed for the mobile. The difference between the two identification levels was considerably smaller for the Swiss than for the mobile French. This suggests that the mobile French were motivated to emphasize their higher valued identity and to distance it from the lower valued one. The Swiss, although to a smaller extent, focused more on the inherited than the achieved identity. This may be because it is their inherited identity that clearly differentiates them from the French mobile individuals, and so fulfills their need of a positive, but also, distinct social identity (Tajfel and Turner, 1986). Indeed, their achieved identity is more malleable and also shared by a portion of the French and it may thus be considered as less important.

### Study 2

In Study 2, we focused on French nationals in order to examine concern for the inherited low-status group at different stages of the social mobility process. We used the same setting as in Study 1, with three modifications. The main modification consisted in distinguishing, among the French working in France, between those who anticipated social mobility by expressing the desire to work in Switzerland in the future (i.e., mobility anticipators) and those who did not (i.e., non-anticipators). Second, the target of the involvement dependent measure was the inherited low-status group (and not the achieved low-status group). Third, we measured prejudice toward the mobile group. The aim was to test if the mere anticipation of social mobility is sufficient to produce a tendency toward self-ingroup distancing, a phenomenon that should be most prominent among the mobile. As predicted in H2, we expected a linear effect of social mobility on concern for the low-status inherited group, showing the highest concern among non-anticipating individuals, a moderated concern among anticipators, and the lowest concern among mobile individuals. In addition, we investigated whether the differentiation of mobility anticipating, mobility non-anticipating, and mobile individuals, revealed different levels of inherited group identification. As predicted in H3, such differences should, in turn, account for the gap in these groups’ ingroup attitudes.

#### Method

##### Participants and procedure

Participants were 216 French nationals (137 women and 79 men, *M*_age_ = 34.54, *SD*_age_ = 10.16, ranging from 20 to 61 years of age) living in border regions of Switzerland. We used the same recruitment procedure as in Study 1. After reporting their nationality, we presented a short introductive text in order to prime the status gap between French nationals working in France (low-status achieved group) and French nationals working in Switzerland (high-status achieved group). Following this, participants indicated the country of their employment. Then, participants proceeded to the measures outlined below in chronological order.

##### Measures

###### Social mobility

We distinguished between three groups of different social mobility stages. Participants who worked in Switzerland were categorized as *mobile* (*n* = 95). Participants who worked in France (*n* = 121) were asked to report the extent to which they would like to work in Switzerland in the future (1 *not at all* to 7 *totally*, *M* = 4.69, *SD* = 2.32). A median-split on the responses to this question (median = 5) provided two subgroups of participants: the mobility *anticipators* (*n* = 58) who expressed strong desire to work in Switzerland (*M* = 6.76, *SD* = 0.43), and the *non-anticipators* (*n* = 63; *M* = 2.78, *SD* = 1.61) who reported a lower desire for mobility. Thus, the mobile and the anticipators can be considered as “psychologically mobile” because they either have, or are considering, to be mobile, whereas the non-anticipators have not.

###### Identification with the inherited and the achieved groups

As in Study 1, participants’ identification with the inherited and the achieved groups was measured with Leach et al.’s (2008) identification scale. Participants were first asked to state their identification with the inherited group (i.e., French people in general; α = 0.93; *M* = 4.38, *SD* = 1.44), and with the achieved group (i.e., workers in France for anticipators and non-anticipators, α = 0.92, *M* = 4.10, *SD* = 1.43; or workers in Switzerland for mobile participants, α = 0.89; *M* = 5.40, *SD* = 1.11).

###### Concern for the inherited group

To assess ingroup concern, we measured participants’ motivation to get involved in actions aimed to improve the situation of French people living in border regions of Switzerland. As in the first study, we used two items indicating support for group involvement in collective action (e.g., “The French should unite and show solidarity with each other to collectively fight against a decline in their standard of living”, *r* = 0.64, *p <* 0.001; *M* = 4.67, *SD* = 1.79), and two items indicating the motivation to get personally involved in collective action (e.g., “I would be willing to get personally involved to improve the economic and social situation of the French in a precarious situation (e.g., pay more taxes)”, *r* = 0.55, *p <* 0.001; *M* = 3.78, *SD* = 1.87). Both constructs were measured with 7-point scales from 1 *not at all* to 7 *totally*.

###### Prejudice toward frontier workers

In addition, we measured participants’ prejudice toward frontier workers with four items. Sample items are: “Because of their special status, frontier workers should pay a solidarity tax to help French people living in border areas and working in France, who are suffering from rising prices (e.g., estate market)” and “Frontier workers only think of their own interest and often forget their origins” (α = 0.71; *M* = 3.68, *SD* = 1.58).

###### Socio-demographic information

Finally, participants reported their gender, their professional status (with in general 62.5% employee, 8.8% entry-level manager, 12% middle manager, 4.6% senior manager, and 12% of missing data), their age, and the subjective status of their occupation (same two items as in Study 1, *r* = 0.36, *p <* 0.001, *M* = 3.86, *SD* = 1.34).

#### Results

##### Preliminary analyses

As in Study 1, we conducted preliminary analyses in order to examine potential socio-demographic differences between the three social mobility groups. A chi-square test showed that gender was not similarly distributed across the three groups, χ^2^(2, *N* = 216) = 10.26, *p* = 0.006, *Cramer’s V* = 0.22. Whilst men and women were equally represented among mobile participants, the sample showed an overrepresentation of women among the non-anticipators (73% of women vs. 27% of men) and the anticipators (72.4% of women vs. 27.6% of men). A further chi-square analysis showed no differences in terms of the professional status between the three groups, χ^2^(6, *N* = 190) = 2.80, *p* = 0.83. An ANOVA testing the effect of the three groups on subjective professional status of the participants’ occupation revealed no significant effect, *F*(2,216) = 2.00, *p* = 0.14, $\eta_{p}^{2}$ = 0.02. Finally, an ANOVA showed an effect of participants’ social mobility stage on age, *F*(2,216) = 6.28, *p* = 0.002, $\eta_{p}^{2}$ = 0.07, revealing that non-anticipators (*M* = 37.75, *SD* = 11.28) were significantly older than anticipators (*M* = 30.47, *SD* = 8.88, *p <* 0.001), and marginally older than mobile participants (*M* = 34.91, *SD* = 9.33, *p* = 0.08). The latter group was also older than the anticipators (*p* = 0.007). Based on these results, we included gender and age as covariates in all the following analyses^2^.

##### Hypotheses testing

###### Concern for the inherited ingroup

In order to test H2, which predicts a linear effect of social mobility, we computed two orthogonal contrasts with the social mobility variable. The first contrast (C1 opposed the non-anticipators of social mobility (i.e., French workers in France who do not wish to work in Switzerland in the future), coded -1, to the mobile (i.e., French workers in Switzerland), coded 1. Anticipators (i.e., French workers in France who wish to work in Switzerland) were coded 0 and were thus situated between the two former groups. The residual contrast (C2) tested differences between the anticipators, coded -2, and the two other groups, the non-anticipators and the mobile, both coded 1. As expected in H2, we predicted a significant effect of C1 but not of C2, thus highlighting a linear effect of our social mobility variable (Judd et al., 2011).

We conducted a repeated-measures ANCOVA with the two orthogonal contrasts as between-participants factors, involvement in collective action (group versus personal involvement) as a within-participant factor, and age and gender (coded women -1 and men 1) as covariates. The findings first showed a main effect of involvement, *F*(1,211) = 165.28, *p <* 0.001, $\eta_{p}^{2}$ = 0.44. Participants reported greater support for group involvement in collective action (*M* = 5.55, *SD* = 1.31), than for personal involvement (*M* = 3.96, *SD* = 1.67). The interaction between the involvement dimensions and C1 was also significant, *F*(1,211) = 4.61, *p* = 0.03, $\eta_{p}^{2}$ = 0.02, but while C1 had a significant impact on the personal dimension of collective action, *t* = -2.62, *p <* 0.01, its impact on the group dimension was not significant, *t* = -0.55, *p* > 0.58. Moreover, we also observed a significant interaction between the involvement dimensions and C2, *F*(1,211) = 6.45, *p* = 0.01, $\eta_{p}^{2}$ = 0.03, showing a significant impact of C2 on the group dimension of collective action, *t* = -2.26, *p* = 0.02, indicating that the anticipators expressed a higher support for the collective action (*M* = 5.84, *SD* = 1.20) from the group than the support of the non-anticipators and the mobile combined (respectively, *M* = 5.46, *SD* = 1.23 and *M* = 5.38, *SD* = 1.40). As C2 was not significant for the personal dimension, the effect of C1 on the personal dimension could be interpreted as linear effect: As expected in H2, the non-anticipators (*M* = 4.40, *SD* = 1.63) were more motivated to get personally involved in collective action than the mobile (*M* = 3.69, *SD* = 1.73), with the anticipators (*M* = 3.92, *SD* = 1.55) situated between these two groups. Finally, the analysis produced a significant interaction between gender and involvement, *F*(1,211) = 4.09, *p* = 0.04, $\eta_{p}^{2}$ = 0.02. However, none of the pairwise comparisons reached significance for this interaction (*p*s > 0.27, $\eta_{p}^{2}$ < 0.006). All other effects were non-significant (*p*s > 0.29, $\eta_{p}^{2}$ < 0.005).

###### Prejudice toward the frontier worker status

We performed an ANCOVA on the prejudice expressed toward frontier workers with the two contrasts as between-participants factors, and gender and age as covariates. The findings revealed a main effect of C1, *F*(1,216) = 41.73, *p <* 0.001, $\eta_{p}^{2}$ = 0.16, but not of C2, *F*(1,216) = 1.44, *p* = 0.23, $\eta_{p}^{2}$ < 0.01. Non-anticipators (*M* = 4.40, *SD* = 1.54) reported more prejudice toward frontier workers than mobile participants (*M* = 2.95, *SD* = 1.28), with anticipators (*M* = 4.09, *SD* = 1.60) situated between these two groups. Consistent with H2, we therefore observed a linear effect of the social mobility variable on the prejudice toward frontier worker status.

###### Identification with the inherited and the achieved groups

**FIGURE 2:**
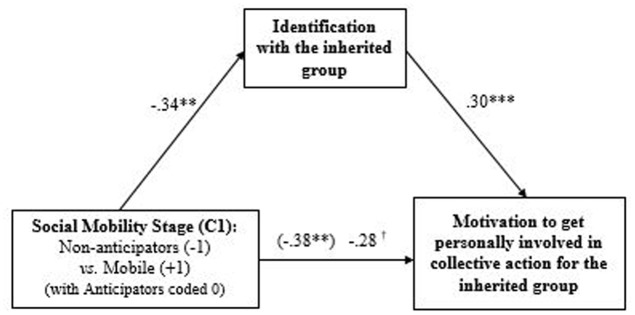
Unstandardized regression coefficients for the relationship between mobility stage (Contrast 1) and the motivation to get personally involved in collective action for the inherited group as mediated by the identification with the inherited group, controlling for C2, age and gender (Study 2). The coefficients in parentheses correspond to the total effect (path c). ^†^*p <* 0.10, ^∗∗^*p <* 0.01, ^∗∗∗^*p <* 0.001.

In order to test H3, which predicts that inherited identification should explain anticipators’ and mobile’s lower ingroup concern (i.e., compared to non-anticipators), we performed PROCESS Model 4 mediation analysis, using 10,000 bootstrapped samples following Hayes’ 2013 recommendations. The model included C1 (non-anticipators versus anticipators versus mobile) as a predictor, personal involvement in collective action as the dependent variable, identification with the inherited group as potential mediator, controlling for C2 (anticipators versus non-anticipators and mobile participants), gender, and age (see full results in **Table 1**). The analysis revealed a significant effect of mobility stage (i.e., C1) on identification with the inherited group (path a: *B* = -0.34, *SE* = 0.12, *p* = 0.007), which in turn was positively associated with personal involvement (path b: *B* = 0.30, *SE* = 0.08, *p <* 0.001). Moreover, identification with the inherited group proved to be a significant mediator, CI 95% [-0.23, -0.02], of the relation between social mobility (C1) and the motivation to get personally involved in collective action for the ingroup. The direct effect of C1 became marginally significant when controlling for the mediator (path c’: *B* = -0.28, *SE* = 0.14, *p* = 0.051). A Sobel test confirmed that the difference between path c and path c’ was significantly different from 0 for the indirect effect of the identification with the inherited group, *z* = -2.16, *p* = 0.03, corroborating the mediating role of the identification with the inherited group. In sum, this study provides evidence of an identity discount strategy in social mobility trajectories. Findings are graphically represented in **Figure 2**.

**Table 1:** Identification with the inherited group as mediator of the relationship between social mobility stages and personal involvement in collective action (Study 2).

Dependent variable (DV)	Personal involvement in CA		
	
Path/Effect	*B*	SE	CI 95%
*a* (C1 → identification)			
Inherited group	-0.34^∗∗^	0.12	[-0.58, -0.09]
*b* (identification →DV)			
Inherited group	0.30^∗∗∗^	0.08	[0.10, 0.44]
*c* (C1 → DV)	-0.28^†^	0.14	[-0.56, -0.001]
*c′* (C1 → DV)	-0.38^∗∗^	0.14	[-0.67, -0.09]
**Covariates**			
*a* (C2 → identification)			
Inherited group	0.11	0.07	[-03, 0.25]
*a* (age → identification)			
Inherited group	0.03^∗∗^	0.01	[0.01, 0.05]
*a* (gender → identification)			
Inherited group	-0.03	0.10	[-0.24, 0.17]
*b* (C2 → DV)	0.04	0.08	[-0.13, 0.20]
*b* (age → DV)	0.004	0.01	[-0.02, 0.03]
*b* (gender → DV)	-0.13	0.12	[-0.36, 0.10]
*c* (C2→ DV)	0.07	0.08	[-0.10, 0.23]
*c* (age → DV)	0.01	0.01	[-0.01, 0.03]
*c* (gender → DV)	-0.14	0.12	[-0.38, 0.10]
**Indirect effects (a × b)**			
Inherited group	-0.10	0.05	[-0.23, -0.02]


*C1, Non-anticipators (coded -1) vs. Mobile (coded 1; with Anticipators coded 0); C2, Anticipators (coded -2) vs. Non-anticipators and Mobile (both coded 1); Estimates are unstandardized; ^†^*p <* 0.10, ^∗^*p <* 0.05, ^∗∗^*p <* 0.01, ^∗∗∗^*p <* 0.001.*

In addition, in order to test the replicability of the identification pattern observed among non-mobile and mobile participants previously observed in Study 1, we performed a repeated-measures ANCOVA with inherited versus achieved identification as a within-participant factor, and the three mobility groups as between-participants factor, controlling for age and gender. Means are displayed in the right panel of **Figure 1**. Results only revealed an interaction between group and identification, *F*(1,211) = 31.5, *p <* 0.001, $\eta_{p}^{2}$ = 0.23. Pairwise comparisons first showed that for achieved identification [*F*(2,211) = 39.34, *p <* 0.001, $\eta_{p}^{2}$ = 0.27] mobile participants identified more strongly with the achieved group than the non-anticipators (*p* = 0.007), and the anticipators (*p <* 0.001), and the anticipators identified less than the non-anticipators (*p <* 0.001). Second, pairwise comparisons showed for inherited identification [*F*(2,211) = 4.32, *p* = 0.01, $\eta_{p}^{2}$ = 0.04] that while anticipators and mobile participants identified to a similar extent (*p* = 0.96), they both identified less with their inherited group than non-anticipators (*p* = 0.01 for the anticipators, and *p* = 007 for the mobile). Finally, we also examined the discrepancy between identification with the inherited group and identification with the achieved group. Findings showed that the mobile identified more with the achieved than with the inherited group, *F*(1,211) = 61.58, *p <* 0.001, $\eta_{p}^{2}$ = 0.23, and that the reverse pattern occurred for the anticipators, *F*(1,211) = 11.6, *p* = 0.001, $\eta_{p}^{2}$ = 0.05. No difference between identification to the achieved and the inherited groups was observed for the non-anticipators, *F*(1,211) = 0.28, *p <* 0.60, $\eta_{p}^{2}$ = 0.001.

##### Discussion

Study 2 investigated the role of the mobility stage on self-distancing from the inherited ingroup, by looking at ingroup attitudes and identification. The novelty of Study 2 is that it took a more fine-tuned perspective on the non-mobile group by distinguishing between those who desired to engage in social mobility in the future and those who did not. Such analysis provided a preliminary insight on the crucial role of the mobility stage on ingroup attitudes and identification.

On the attitudinal dimension, we observed different effects of social mobility on the support for group involvement in collective action and on the motivation to get personally involved in it. First, results revealed the unexpected effect that anticipators of social mobility expressed a greater support for group involvement in collective action compared to the two other groups aggregated. Such finding highlights the particular dissatisfaction of anticipators regarding the fate of their inherited membership. In line with our expectations, results on personal involvement in collective action further revealed that anticipators preferred to focus on their personal trajectory in order to improve their chances to enhance their social identity rather than to join the group in its claim. This result is consistent with Taylor and McKirnan’s (1984) model of social mobility stages arguing that low-status group members would only act collectively if they had failed to individually mobilize. Indeed, as predicted by H2, even if anticipators were more motivated to get personally involved compared to the mobile, they were less motivated compared to the non-mobile, preferring to focus on an individualistic strategy to improve the value of their social identity. Consistent with previous evidence in the literature showing a negative impact of the social mobility process on ingroup concern (Derks et al., 2011a,b, 2015; Kulich et al., 2015), mobile individuals showed the weakest levels of personal involvement. Finally, moving a step further, the present findings demonstrated that the mere anticipation of social mobility is sufficient for triggering a decrease in ingroup concern. We thus argue that experiencing the socialization process of social mobility *per se* is not a necessary condition for lower ingroup concern, but that imagining the possibility to be socially mobile, thus a purely psychological process, is sufficient to engage in attitudinal change.

In this study, we also assessed prejudice toward the mobile group. In parallel to what was observed on ingroup concern, the findings showed that prejudice toward frontier workers was also contingent on the social mobility stage. Again, we observed a linear effect of the social mobility, such that non-mobile participants expressed higher prejudice toward frontier workers than mobile individuals, with the anticipators of social mobility situated between these two groups. Although non-mobile participants had to evaluate their most direct and relevant outgroup, individuals who anticipated social mobility expressed less prejudice toward this group, compared to the non-anticipators. Consistent with Merton’s theorization concerning anticipatory socialization, these findings highlight the positive orientation individuals develop toward an outgroup they aspire to belong to (Merton, 1968). Moreover, the fact that non-anticipators have meanwhile demonstrated a higher level of prejudice toward frontier workers may be related to the previous literature investigating the reaction toward deviance, and particularly the “black sheep effect” (Marques et al., 1988; Pinto et al., 2010). According to this literature, deviance tends to be more severely punished when it comes from an ingroup member than when it comes from an outgroup member. Individuals indeed perceive the deviant’s behavior as threatening to the identity of the ingroup. By the rejection of this behavior and its actor, they reaffirm the ingroup’s standards and contribute to the longevity of the group. Thus, it is not surprising that non-anticipators had unfavorable attitudes toward frontier workers who are ultimately perceived as betrayers, preferring to improve their own status while the whole group continues to suffer from inferior conditions (Blair and Jost, 2003).

As for group identification, novel insights were obtained through the distinction between non-anticipators and anticipators in the non-mobile group. Indeed as expected in H3, the linear decrease of ingroup concern observed throughout social mobility stages was accounted for by a lower identification with the inherited group. Such a finding suggests an identity discount strategy, as derived from SIT assumptions. This strategy points to individuals who distance themselves from their inherited low-status ingroup on both the attitudinal and the identification dimensions (Ellemers, 2001). Extending past research (e.g., Derks et al., 2011b, 2015; Kulich et al., 2015), the findings from Study 2 illustrate the willingness of individuals to increase their chances to attain a better valued social identity through individual mobility, despite the fact that they are unable to actually part with their low-status membership. Moreover, by revealing similar levels of ingroup identification among anticipators and mobile individuals (even though these two groups still differed in their ingroup concern), our data support the idea that ingroup identification plays a crucial role in the process of social mobility (Tajfel and Turner, 1986; Ellemers, 2001). This is also in line with Taylor and McKirnan’s (1984) claim that members of low-status groups who perceive themselves as competent, and so as non-prototypical of their low-status membership, will try everything possible to dissociate themselves from this group.

Consistently, Study 2 revealed the same identification pattern for the mobile French as in Study 1. The mobile identified more with their higher valued achieved group than with their lower valued inherited group. As for the non-mobile individuals, their identification pattern strongly depended on their desire to engage in social mobility. Indeed, those who wished to be socially mobile were less identified with both the inherited and the achieved groups compared to the non-anticipators. These results thus highlight the conflict anticipators may feel between their desire to improve their condition and their actual low-status memberships (Lenski, 1966). Moreover, the non-anticipators were more identified with the inherited group compared to the mobile and the anticipators. Thus, the absence of a difference in inherited identification between non-mobile and mobile participants in Study 1, as well as in Kulich et al. (2015), may have been due to the fact that all non-mobile individuals were treated as one group, thereby mixing two groups (anticipators and non-anticipators) of very different identification patterns. In support of this reasoning, the marginal difference observed in Study 1 between inherited and achieved identification became significant only for the anticipators in Study 2. In sum, it seems that no distance in the identification occurs for non-mobile non-anticipators but a clear motivation to distance between the two arises for anticipators.

The correlational nature of the present research limits the interpretation of causal relationships between social mobility, attitudes and identification. This study only shows results from anticipators and mobile individuals but not the actual process of people who move from the anticipator to the mobile stage. Thus, at least two different mechanisms could be responsible for the patterns observed for anticipators. First, they may start to *dis*identify from the unwanted achieved group in order to replace it by a higher identification with the new high-status group as soon as they actually successfully engage in social mobility. Interestingly, we observed in line with this idea that anticipators showed in Study 2 the same identification with the inherited group as the mobile participants. As previously discussed, this may describe a psychological strategy through which anticipators are adapting to a potential social mobility rather than behaving like other non-mobile individuals. According to Sidanius and Pratto (1999), such individual orientation rests on meritocratic beliefs, which conceptualize as legitimating myths that contribute to protect the social hierarchy by valuing individualistic behavior and strengthening the unequal treatment of members of the two groups. Moreover, this is also in line with the theorization of Merton (1968) and the results we observed on the attitudes toward frontier workers in Study 2. Second, an alternative explanation that cannot be completely discarded is that anticipators and mobile individuals differ from non-mobile due to previous experiences or socialization processes. Controlling for all the possible differences in socialization, attitudes, and employment histories that may exist between the three investigated French groups is an important but difficult task. Future research should thus aim at experimentally manipulating social mobility in order to investigate its impact on attitudes and identification. Longitudinal studies could also be informative as they would allow to assess actual changes in attitudes and (dis)identification patterns.

In addition, further research is needed to determine the extent to which the present findings in ingroup concern can be generalized to broader intergroup attitudes, such as ingroup bias and prejudice expression. Indeed, the measures we used in this research were targeting the personal interests of individuals (e.g., the reduction of the costs of life in the French border regions of Switzerland), thus maximizing the differences between the three groups of participants.

Another limitation of this study is its incapacity to provide insights concerning the consequences of such identity discount strategy on the quality of the relationship mobile individuals maintain with their inherited group members. Indeed, it is reasonable to think that by being simultaneously lowly identified with the inherited ingroup and expressing lower concern for it, mobility anticipators take the risk of being judged as disloyal (Blair and Jost, 2003). This may in turn motivate ingroup members to devalue them as a way to punish the deviance and at the same time to reaffirm the norm. Paralleling this idea, research demonstrates that when women attain high-responsibility positions in the workplace, meaning that they successfully achieved social mobility in a male-dominated domain, they are perceived as less communal than women in general (see for example Rudman and Glick, 2001). Research conducted by Heilman and Okimoto (2007) illustrated that unexpected (i.e., gender incongruent) competence demonstration of agentic women led to punishment in hiring procedures. However, by adding information reaffirming the communality of the female candidates (i.e., by emphasizing their mother status), the selective bias against agentic women decreased as they appeared more stereotypical. Consistently, Phelan et al. (2008) showed that whereas perceived competence was the most important factor predicting the selection of candidates in hiring procedures, the criteria of selection shifted when they came to concern agentic female candidates: Rather than being evaluated based on their competence, they were evaluated based on their social skills, thus being punished if they did not live up to expectations that women should be socially skilled.

## General Discussion

This research was aimed at providing further insights about the deleterious impact of upward mobility on attitudes toward the inherited low-status ingroup. In Study 1, we observed that socially mobile individuals expressed negative attitudes toward non-mobile group members, simultaneously with a higher identification with their achieved group. In parallel, inherited identification was not different between these groups, a pattern that illustrates their desire to assimilate to the high-status group. On the one hand, mobile individuals have the willingness to improve their social condition, but on the other hand they are bound by the inevitable membership in a low-status group. Of interest, Van Laar et al. (2014) showed that members of low status groups and those of high social status groups do not offer support for mobile individuals under the same conditions. On the one hand, high-status group members appear to be more sensitive to the behavioral dimension than the affective one, preferring mobile individuals who did not behave as a prototype of their low-status ingroup regardless of their identification with this group. On the other hand, low-status group members prefer mobile individuals who keep a strong identification with their ingroup, regardless of their level of behavioral prototypicality, or their competence (Campos et al., 2016). In light of these recent works, the negative ingroup attitudes expressed in the present research by the French mobile together with the expression of affective proximity with this group can be apprehended as a strategy to increase their integration in the high-status group, without breaking the affective ties they have with their inherited ingroup.

Extending these findings to actual social mobility, Study 2 further examined the impact of the willingness to engage in social mobility among individuals who have been so far non-mobile. Of interest, results showed that the mere anticipation of social mobility was sufficient to produce a lower concern for the ingroup. In addition, this tendency appeared to be due to an identity discount strategy (Tajfel and Turner, 1986; Ellemers, 2001). Indeed, we observed that the lower ingroup concern expressed by the mobile and, to a lesser extent by the anticipators, was accounted for by their lower identification with their inherited group. Therefore, by distinguishing between non-mobile individuals based on their willingness to undertake mobility, we provided further insights about the process of social mobility. Despite recent findings illustrating the maintenance of identification with the low-status group and its coexistence with high levels of identification with the high-status group (Kulich et al., 2015), our research rather emphasizes an identity discount strategy as a privileged way for mobile individuals to cope with their status-inconsistent identity configuration. Nevertheless, little is known about the mechanisms leading social mobility anticipators, as well as mobile individuals to engage in such a coping strategy. Further investigations are thus needed in order to unravel such social dynamics and to identify precisely the conditions favoring the expression of such an identity discount.

In summary, it seems that individuals anticipating upward mobility follow the principle “I want – therefore I am”. Indeed, they already start to dissociate from their low-status group, not only through their (more negative) attitudes toward it, but also through their level of identification with the inherited group, as characterized by the identity discount strategy uncovered in this research. This attitudinal and identity discounts allow them to reduce the dissonance they may experience as a result of the asymmetrical statuses of their different groups’ membership. The felt dissonance could even be stronger than the one experienced by mobile individuals, because of the coexistence of their motivation to improve their condition and the unescapable nature of their inherited membership.

## Ethics Statement

This study was carried out in accordance with the recommendations of ‘Ethical code concerning research at the Faculty of Psychology and Educational Sciences at the University of Geneva, Ethical Commission’ with written informed consent from all subjects. In all studies, participants ticked a box before starting the survey and after finishing it, indicating their informed consent and agreement to use their responses for research purposes. The protocol was approved by the ‘Ethical Commission of the Faculty of Psychology and Educational Sciences at the University of Geneva’.

## Author Contributions

MC, CK, VI, and FL-C contributed to the study design. MC collected the data. MC performed the data analysis and interpretation under the supervision of CK, VI, and FL-C. MC drafted the manuscript, and CK, VI, and FL-C provided revisions.

## Conflict of Interest Statement

The authors declare that the research was conducted in the absence of any commercial or financial relationships that could be construed as a potential conflict of interest.

## Footnotes

1 Controlling for gender, professional status, and subjective social status in our analyses on concern for the low-status achieved group and identification did not affect the pattern of results, thus we do not report the details here.

2 Again, controlling for professional status and subjective social status in our analyses on ingroup concern, prejudice toward the frontier workers, and group identification did not affect the pattern of results, thus we have not reported the details here.
